# Secretion of the endoplasmic reticulum stress protein, GRP78, into the BALF is increased in cigarette smokers

**DOI:** 10.1186/s12931-017-0561-6

**Published:** 2017-05-02

**Authors:** Mark O. Aksoy, Victor Kim, William D. Cornwell, Thomas J. Rogers, Beata Kosmider, Karim Bahmed, Carlos Barrero, Salim Merali, Neena Shetty, Steven G. Kelsen

**Affiliations:** 1 Department of Thoracic Medicine and Surgery, Temple University School of Medicine, Philadelphia, PA 19140 USA; 2 761 Parkinson Pavilion, Temple University Hospital, 3401 N. Broad St., Philadelphia, PA 19140 USA; 3 Department of Physiology, Temple University School of Medicine, Philadelphia, PA 19140 USA; 4 Center for Inflammation, Translational and Clinical Lung Research, Temple University School of Medicine, Philadelphia, PA 19140 USA; 5 Temple University School of Pharmacy, Philadelphia, PA 19140 USA

**Keywords:** Oxidant stress, GRP78, Cigarette smoke, COPD, Histone deacetylase, Biomarker, Human, Lung

## Abstract

**Background:**

Identification of biomarkers of cigarette smoke –induced lung damage and early COPD is an area of intense interest. Glucose regulated protein of 78 kD (i.e., GRP78), a multi-functional protein which mediates cell responses to oxidant stress, is increased in the lungs of cigarette smokers and in the serum of subjects with COPD. We have suggested that secretion of GRP78 by lung cells may explain the increase in serum GRP78 in COPD. To assess GRP78 secretion by the lung, we assayed GRP78 in bronchoalveolar lavage fluid (BALF) in chronic smokers and non-smokers. We also directly assessed the acute effect of cigarette smoke material on GRP78 secretion in isolated human airway epithelial cells (HAEC).

**Methods:**

GRP78 was measured in BALF of smokers (S; *n* = 13) and non-smokers (NS; *n* = 11) by Western blotting. GRP78 secretion by HAEC was assessed by comparing its concentration in cell culture medium and cell lysates. Cells were treated for 24 h with either the volatile phase of cigarette smoke (cigarette smoke extract (CSE) or the particulate phase (cigarette smoke condensate (CSC)).

**Results:**

GRP78 was present in the BALF of both NS and S but levels were significantly greater in S (*p* = 0.04). GRP78 was secreted constitutively in HAEC. CSE 15% X 24 h increased GRP78 in cell-conditioned medium without affecting its intracellular concentration. In contrast, CSC X 24 h increased intracellular GRP78 expression but did not affect GRP78 secretion. Brefeldin A, an inhibitor of classical Golgi secretion pathways, did not inhibit GRP78 secretion indicating that non-classical pathways were involved.

**Conclusion:**

The present study indicates that GRP78 is increased in BALF in cigarette smokers; that HAEC secrete GRP78; and that GRP78 secretion by HAEC is augmented by cigarette smoke particulates. Enhanced secretion of GRP78 by lung cells makes it a potential biomarker of cigarette smoke–induced lung injury.

## Background

Identification of biomarkers of cigarette smoke – induced lung damage and early COPD is an area of intense interest [1–5]. Chronic cigarette smoking, the major cause of COPD, induces oxidant stress and the accumulation of misfolded, non-functional proteins in the endoplasmic reticulum (ER) of lung cells [6–8].

Glucose regulated protein of 78 kD (i.e., GRP78), an ER chaperone required for the processing and transport of key lung proteins (e.g., CFTR and surfactant protein C) [9–11] is up-regulated in the lung by cigarette smoke [8] and has been reported as a potential biomarker for COPD [2, 12]. GRP78 levels are increased in the serum of subjects with COPD and correlate with both FEV1 and the severity of emphysema [12].

We have speculated that lung structural cells are the source of the increase in serum GRP78 in COPD. If so, GRP78 may also be secreted into the airway surface lining and, hence, may be increased in the BALF of chronic smokers.

Accordingly, several approaches were taken in the present study. First, we determined if GRP78 is both present in human BALF and increased in chronic cigarette smokers. Second, we directly examined the acute effects of cigarette smoke on the secretion of GRP78 by cultured human airway epithelial cells (HAEC).

The present study indicates that BALF levels of GRP78 are increased in cigarette smokers; that GRP78 is secreted constitutively by HAEC; and that the volatile but not the particulate material i.e., the cigarette tar, augments GRP78 secretion.

## Methods

### Study population and BALF collection

Studies were performed on healthy never smokers (NS, *n* = 11) and healthy, chronic smokers (S, *n* = 13). Subjects were studied as stable outpatients. Measurements of FEV1, FVC and FEV1/FVC were obtained prior to bronchoscopy using a spirometer which met American Thoracic Society standards.

Bronchoscopy was performed as previously described [13]. BALF samples were obtained by infusion of 200 ml saline into the right middle lobe. The return was gauze - filtered, centrifuged at 250 g × 15 min, aliquoted and stored at −80 °C until used. Aliquots were subsequently concentrated 40 X using Amicon Ultra filters (30 KD cutoff) (EMD Millipore Corp., Billerica, MA) by centrifugation at 3,200 g for 20 min. Concentrated samples were stored at −80 ° C. GRP78 levels in BALF were then assessed by Western blotting (see below). 20 μl of protein was loaded. Human serum albumin in BALF was quantitated by ELISA (R&D Systems, Minneapolis, MN, #DY1455).

### Human airway epithelial cell (HAEC) culture

HAEC were obtained by bronchoscopy from healthy, non-smoking volunteers by airway brushing as previously described [13]. Cells were cultured on collagen matrix to 80% confluence in defined medium (BEGM, Lonza, Walkersville, MD). Prior to study, cells were washed with a GRP78-free medium, i.e., BEGM devoid of the bovine pituitary extract (BPE) supplement.

### Cigarette smoke exposure

Cells were separately exposed to two forms of cigarette smoke material, the volatile phase, termed cigarette smoke extract (CSE), and the particulate material i.e., tar, termed cigarette smoke condensate (CSC). CSE was prepared as previously described [8] by bubbling smoke from 2 unfiltered research cigarettes (2R4, University of Kentucky) into 10 ml of BEGM in a parafilm-sealed centrifuge tube at a flow rate of 50 ml/min. After a 10 min equilibration, the medium was centrifuged (3,000 rpm × 10 min) to remove particulate matter, sterile-filtered (0.2 μm), diluted to 15% by volume in BEGM and applied to the cells within 30 min of preparation. CSC (Murty Pharmaceuticals, Lexington, KY), dissolved in DMSO, was diluted to a final concentration of 150 μg/ml in BEGM. Cells were treated with CSE (15%), CSC (150 μg/ml), or vehicle (DMSO) for 24 h.

Cell - conditioned medium was removed, centrifuged (3,000 rpm for 10 min) and concentrated on Amicon Ultra filters as described above. Cells were washed, removed by scraping and then lysed by 4 cycles of freeze (−80 °C)/thawing. Cell lysates were centrifuged (12,000 rpm × 5 min) and total protein was determined by DC assay (Bio-Rad, Hercules, CA). Samples were stored at −80 °C until used for Western blotting (see below).

### Western blot analysis

Samples (15 μg total protein) were separated on 10% SDS-PAGE, transferred to nitrocellulose membranes in CAPS buffer, blocked with 5% non-fat milk, 1% BSA in Tris-buffered saline solution / 0.05% Tween-20 (pH 7.6), and probed overnight at 4 °C for GRP78 (BD Biosciences, San Jose, CA, Cat#610979) (1:1,000 dilution).

Membranes were then incubated with mouse anti-rabbit Hrp-conjugated secondary antibody (Santa Cruz Biotechnology, Santa Cruz, CA, Cat# sc-2004) for 1 h at room temperature (1:10,000) and then exposed on film (Super Signal West Pico Chemiluminescent Substrate, Thermo Scientific, Rockford, IL, Cat#34077). Blots of cell lysates were reprobed for GAPDH (sc-25778) (1:400), a housekeeping gene used as a loading control. Blots of cell-conditioned medium were reprobed for β-actin (sc-8432) (1:400), a non-secreted cytoplasmic protein, which served as a marker for cell lysis. Blots of cell-conditioned medium and cell lysates were reprobed for fibronectin (sc-18825) (1:500), used as a positive control for the effect of brefeldin A on GRP78 secretion. Blots of cell lysates were reprobed with an acetylated-lysine antibody (Cell Signaling Technology, Danvers, MA) (1:1,000) to assess protein acetylation. All reprobed blots were incubated overnight with primary antibody at 4 °C. Recombinant GRP78 (Stress Marq, Victoria, BC, #SPR-119A) was used to quantitate GRP78 immunoblots.

Films were scanned and protein bands of interest quantitated by densitometry using Photoshop CC2015 (Adobe Corp., San Jose, CA).

### Potential mechanism(s) underlying GRP78 secretion

Cigarette smoke affects several important cellular processes which alter GRP78 expression and metabolism in lung cells and, hence, may stimulate GRP78 secretion. For example, cigarette smoke induces the unfolded protein response, a multiple gene program to relieve ER stress due to accumulation of unfolded proteins in the ER [8]. Cigarette smoke also inhibits histone deacetylase activity [14]. Accordingly, we used standard pharmacological probes to mimic these two conditions and examined the effects on GRP78 secretion. Thapsigargin (TG; 1 μM), the classic ER stress inducer which depletes the ER of calcium [15], was used to examine the role of ER stress. Histone deacetylase (HDAC) activity was inhibited using a cocktail of classic pharmacologic inhibitors (trichostatin A (2 μM), MS275 (2 μM) and vorinostat (2 μM)). This combination of inhibitors induces GRP78 acetylation by inhibiting all 4 classes of HDAC inhibitors including the ER HDACs 1, 2, 3 [16–18].

HAEC were treated for 24 h with either TG; HDAC inhibitors; or vehicle (0.1% ethanol plus 0.2% DMSO).

In separate experiments designed to examine the mechanistic pathways underlying secretion, HAEC were pre-treated for 3.5 h with the inhibitor of ER to Golgi transport, brefeldin A (BF-A, 0, 5, 15 or 25 μg/ml) then treated for 24 h with TG (1 μM) + HDAC inhibitors (vorinostat + MS275 + trichostatin A, all 2 μM) or vehicle (DMSO 0.2% and ethanol 0.1%). Fibronectin, which is secreted from HAEC via the classical Golgi pathway [19], was used as a positive control.

### Data analysis

Group data are expressed as mean ± SEM. Statistical significance of differences in group mean was determined by 1-way ANOVA on ranks followed post-hoc by Student’s *t*-test, or by Foster’s Exact Test. Significance was set to *p* < 0.05.

## Results

### GRP78 is increased in BALF from cigarette smokers

Demographic characteristics, smoking history and spirometry of the study population are shown in Table 1. The subject groups were similar in age, gender and BMI. FEV1, FVC and FEV1/FVC were not significantly different between groups.

**Table 1:** Study Population (Mean ± SE)

	Never–Smoker	Smoker	*p* Value
	(*n* = 11)	(*n* = 13)	
Gender M:F	8:3	4:9	0.24
Age (years)	51±3	50±2	0.91
BMI	30±2	31±1	0.83
Smoking History (pack-years)	---	24±3	---
Active : Ex-Smokers	---	13:0	---
FEV1 (% predicted)	92±3	93±4	0.89
FVC (% predicted)	92±4	97±4	0.44
FEV1/FVC (%)	101±2	96±2	0.12

Gender *p* value determined by Fisher Exact Test, all others by One Way ANOVA

**Fig. 1:**
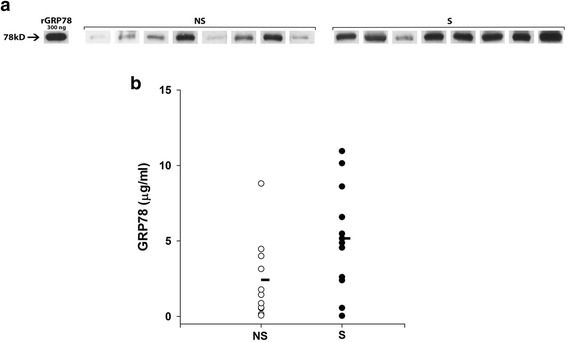
**a** and **b.** GRP78 is present in BALF from smokers and never smokers. **a** Western blot showing GRP78 in BALF samples from never smokers (NS) and smokers (S). Recombinant GRP78 (rGRP78) is shown for quantitation (300 ng). **b** Individual values of BALF GRP78 in never smokers (NS, *n* = 11) and healthy smokers (S, *n* = 13). Mean GRP78 (horizontal bars) was significantly greater in smokers versus never smokers (*p* = 0.04)

**Fig. 2:**
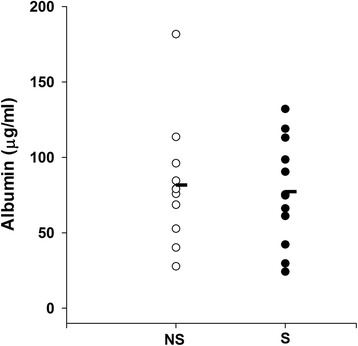
Individual values of BALF albumin in never smokers (NS, *n* = 11) and healthy smokers (S, *n* = 13). Mean albumin (*horizontal bars*) was not significantly different between smokers and never smokers (*p* = 0.34)

GRP78 was present in BALF from both non-smokers and smokers (Fig. 1a), but varied widely in both groups. GRP78 was significantly greater in smokers (5.2 ± 1.0 SE μg/mL) compared to never smokers (2.4 ± 0.9 SE μg/mL) (*p* = 0.04; Fig. 1b). In contrast, albumin levels in BALF were not significantly different in smokers (77.2 ± 10.1 SE μg/mL) and never smokers (81.7 ± 12.4 SE μg/mL) (*p* = 0.34) (Fig. 2).

### Cigarette smoke stimulates GRP78 secretion in HAEC

**Fig. 3:**
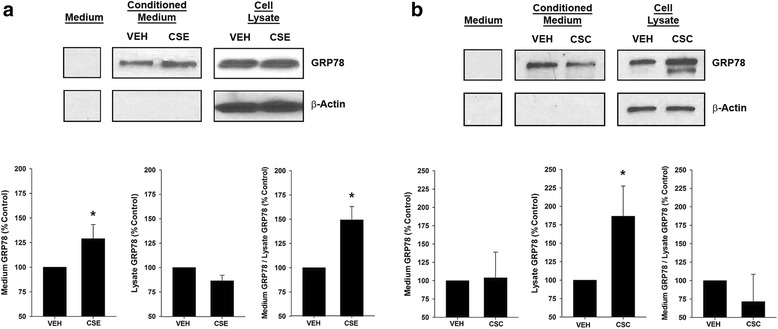
GRP78 secretion by HAEC is increased by cigarette smoke extract (CSE), but not cigarette smoke condensate (CSC). **a** Representative Western blot for vehicle control (VEH) or CSE (15% for 24 h) – treated cells. GRP78 was present in cell-conditioned medium but not medium alone. CSE increased GRP78 in the medium but not the lysate. β-actin, a highly expressed cytosolic protein, was not detected in the cell-conditioned medium. Group mean data of 5 CSE experiments showing GRP78 values normalized to vehicle control in cell-conditioned medium; in cell lysate; and the ratio of medium GRP78 relative to cell lysate GRP78. CSE significantly increased GRP78 in the medium and the ratio of medium GRP78/lysate GRP78 (*p* = 0.008 for both). **b** Representative Western blot for vehicle control or CSC (150 μg for 24 h) – treated HAEC. CSC increased GRP78 in the lysate but not the medium. β-actin was not detected in the cell-conditioned medium. Group mean data of 4 CSC experiments. CSC significantly increased lysate GRP78 (*p* = 0.03) without affecting GRP78 in the medium or the ratio of medium GRP78/lysate GRP78 (*p* > 0.1)

As expected, GRP78 was present in cell lysates (Fig. 3), but was also detected in cell-conditioned medium from HAEC at 24 h (Fig. 3) (*n* = 4–5) and as early as 8 h (data not shown).

Exposure of cells to CSE 15% × 24 h significantly increased GRP78 in the cell-conditioned medium (*p* = 0.008) without affecting GRP78 in cell lysates (Fig. 3a; *n* = 5). As a result, CSE treatment increased the ratio of medium GRP78 / lysate GRP78 which was taken as an indication of GRP78 secretion (*p* = 0.008). In contrast, treatment with CSC (150 μg / mL for 24 h) increased GRP78 in the cell lysate (*p* = 0.03) without affecting the level of GRP78 in the cell–conditioned medium or the medium GRP78 / lysate GRP78 ratio (Fig. 3b; *n* = 4).

CSE and CSC treatment did not affect HAEC number (*p*>0.05). For example, cell number in CSE and CSC experiments was 104 ± 20% SE and 109 ± 22% SE of vehicle control, respectively (*p*> 0.75 for both).

Moreover, β-actin, a marker of cell lysis, was not detected in the cell-conditioned medium (Fig. 3a, b).

### ER stress and HDAC inhibition induce GRP78 secretion

Since cigarette smoke induces ER stress and inhibits histone deacetylase activity, two cellular processes which augment GRP78 metabolism, we examined the effects of the ER-stress inducer thapsigargin (TG) and of HDAC inhibitors on GRP78 secretion.

Of interest, TG treatment increased GRP78 in both cell lysates and cell-conditioned medium (*p=*0.01 and *p=*0.03, respectively) (Table 2). Moreover, the relative increase in secreted GRP78 in the medium (~400%) exceeded the increase in cellular GRP78 (~150%). Accordingly, the ratio of medium GRP78/cell lysate GRP78 was increased by TG treatment (*p=*0.03) (Table 2).

**Table 2:** Effect of Thapsigargin and HDAC Inhibitors on GRP78 Secretion (Mean ± SE, n = 5–7 expts.)

Treatment	Medium GRP78	Lysate GRP78	Medium/Lysate
	% Control	% Control	% Control
HDAC Inhibitors	199 ± 22^++^	97 ± 8	203 ± 22^++^
Thapsigargin	357 ± 102*	139 ± 10^+^	280 ± 94*

Human airway epithelial cells (HAEC) were treated with thapsigargin (TG, 1 μM); HDAC inhibitors (vorinostat + MS275 + trichostatin A, all 2 μM); or vehicle control (DMSO 0.2% and ethanol 0.1%) for 24 h. * *p* = 0.03; ^+^ *p* = 0.01; ^++^ *p* = 0.006

**Fig. 4:**
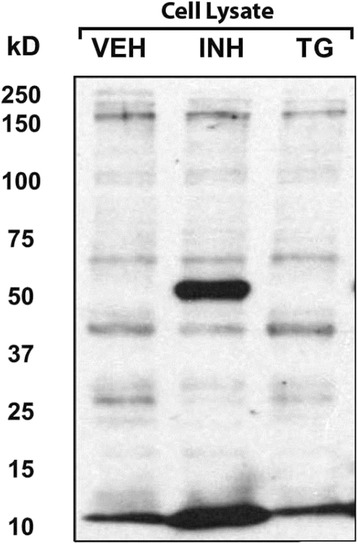
HDAC inhibitors acetylate proteins in cell lysates of HAEC. Representative Western blot (1 of 4 experiments) showing cell lysates probed with the acetyl-lysine antibody. Note that the 50 kD and 10 kD bands were increased by HDAC inhibitor treatment (INH). In contrast, thapsigargin treatment (TG) had no effect on the band pattern when compared to vehicle control (VEH)

Treatment with HDAC inhibitors increased the concentration of acetylated proteins in the cell lysate, in particular at 50 and 10 kD (Fig. 4). HDAC inhibitor treatment increased GRP78 in cell-conditioned medium (*p=*0.006) but not in cell lysates (Table 2). Consequently, the medium GRP78/lysate GRP78 ratio increased with HDAC inhibitors (*p=*0.006; Table 2).

TG and HDAC inhibitors did not affect HAEC number. For example, cell number in TG and HDAC inhibitor experiments was 85 ± 15% SE and 89 ± 15% SE of vehicle control, respectively (*p>* 0.45 for both). Moreover, β-actin, a marker of cell lysis, was not detected in the cell-conditioned medium.

### Secreted GRP78 uses non-classical mechanism

**Fig. 5:**
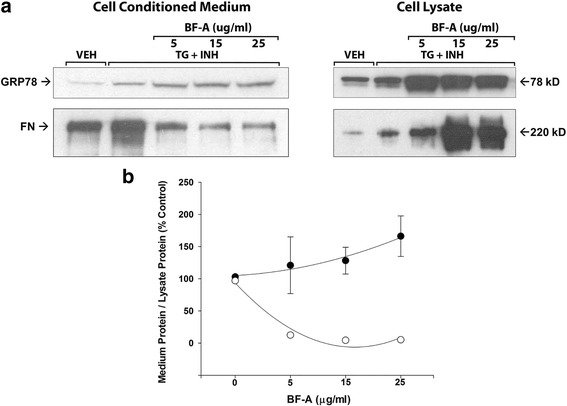
**a** and **b** Brefeldin A (BF-A) inhibits fibronectin (FN) without affecting GRP78 secretion by HAEC. Cells were pre-treated for 3.5 h with BF-A (0, 5, 15 or 25 μg/ml) then treated for 24 h with TG (1 μM) + HDAC inhibitors (vorinostat + MS275 + trichostatin A, all 2 μM) or vehicle (DMSO 0.2% and ethanol 0.1%). **a** Representative Western blot showing that BF-A dose-dependently inhibited fibronectin but not GRP78 secretion. One experiment of 2. **b** Group mean data ± SEM of 2 experiments showing ratio of medium GRP78/cell lysate GRP78 (closed symbols) and medium FN/cell lysate FN (open symbols). Values were normalized to vehicle control. Error bars for FN are smaller than the symbols. The decrease in FN ratio was significant (*p* = 0.03 by ANOVA)

To determine if GRP78 is secreted via the classical ER - Golgi to plasma membrane pathway, cells were treated with brefeldin A [20]. Brefeldin dose-dependently decreased fibronectin (FN) in cell conditioned - medium but increased it in cell lysates (Fig. 5), such that the medium FN / lysate FN ratio decreased (*p* = 0.03) (Fig. 5B). In contrast, brefeldin increased GRP78 levels in both cell-conditioned medium and cell lysates (Fig. 5), without affecting the medium GRP78/lysate GRP78 ratio (*p* > 0.05) (Fig. 5B).

## Discussion

Identification of biomarkers of lung damage and early COPD is an area of active interest [1–5]. GRP78 is a member of the heat shock 70 protein super family [21] and a key ER chaperone [10]. GRP78 processes and transports key lung proteins like the surfactant proteins and cystic fibrosis transmembrane regulator (CFTR) in alveolar type II cells and airway epithelial cells, respectively, and is required for normal lung function [22–24]. In fact, mutations in GRP78 which either impair its chaperone function or decrease its expression, reduce surfactant content in lamellar bodies, induce ER and oxidant stress in the lung, induce alveolar cell apoptosis, and cause neonatal death shortly after birth [22, 23].

We [25] and others [26] have previously shown that GRP78 is highly expressed in airway and alveolar type II epithelial cells in the adult lung. Its expression is increased still further in these cells by cigarette smoke exposure [8, 25]. Moreover, GRP78 is increased in the plasma of subjects with COPD, and levels correlate with the severity of lung damage [12].

Our results indicate for the first time that GRP78 is detectable in BALF of normal subjects and that GRP78 levels in BALF are significantly greater in active smokers. The presence of GRP78 in BALF is likely explained by our observations that cultured HAEC constitutively secrete GRP78. The heightened levels of BALF GRP78 observed in chronic cigarette smokers appear to be explained by our observation that GRP78 secretion by lung epithelial cells is stimulated by the volatile materials in cigarette smoke.

That GRP78 is secreted by HAEC is strongly supported by several observations. First, GRP78 was present only in cell-conditioned medium, not in medium alone. Second, the ratio of GRP78 in the medium relative to the cell lysate was increased by CSE, TG and the HDAC inhibitors. Third, there was no cell lysis as reflected by stability of cell number and no detectable β-actin in the medium. Fourth, our findings are consistent with prior observations of GRP78 secretion [27]. In particular, GRP78 is secreted into synovial fluid and oviductal fluid [28, 29]. Our results suggest that lung structural cells in which GRP78 is abundant, such as airway epithelial cells, are the likely source of heightened GRP78 in the BALF in chronic smokers and in the blood in COPD.

ER stress is increased and HDAC activity is reduced in the lung by cigarette smoke exposure [8, 14, 25, 30, 31]. The present study employed pharmacological probes to induce ER stress and reduce HDAC activity. These interventions promoted secretion of GRP78 by HAEC suggesting that cigarette smoke augments GRP78 secretion by activating these cell processes.

GRP78 is not secreted via the classic ER to Golgi pathway since its secretion is not blocked by the classic ER to Golgi transport blocker, brefeldin A [32, 33]. Hence, GRP78, unlike fibronectin [19], utilizes an unconventional protein secretory pathway (UPS) [32–34]. A variety of cellular transport mechanisms typically activated by cell stress or inflammation, mediate UPS [33]. For example, cytosolic proteins including integrins, cytokines (e.g., IL-1β) and growth factors (e.g., fibroblast growth factor 1 and 2) may exit the cell as cargo in endosomal or autophagic vacuoles or undergo direct secretion through cell membrane pores. Typically, these proteins lack a signal peptide and/or transmembrane domain. GRP78 is present in the cytoplasm as well as the ER; forms alternatively spliced transcripts which may or may not contain a signal peptide; and can exist as a transmembrane protein [35]. Accordingly, one or more of the several possible UPS mechanisms mentioned above may apply.

GRP78 is a “moonlighting protein” [36] that can be redistributed to other locations in the cell where it exerts multiple functions potentially important in the pathogenesis of cigarette smoke-induced lung disease. For example, at the cell surface, GRP78 complexes with both extracellular proteins and specific cell surface – anchored proteins thereby acting as a regulator of cell signaling pathways implicated in cell morphology, survival/proliferation, apoptosis and fibrogenesis [35, 37]. Specifically, GRP78 acting at the cell surface affects signaling via the PI3K/AKT, SMAD, MAPK / Src and caspase pathways [35, 37–39]. Of interest, GRP78 appears to play a role in inflammation. For example, knock-down of GRP78 inhibits nuclear translocation of NF-kβ in LPS – treated cultured human airway epithelial cells [40]. In addition, extracellular GRP78 acting through as yet unidentified receptors exerts anti-inflammatory and immunomodulatory effects and has been included as a member of the resolution associated molecular patterns (RAMPs) class [41]. For example, in blood monocytes, extracellular GRP78 augments release of IL-10, IL-1 beta receptor antagonist and soluble TNF receptor II [42]. Moreover, in dendritic cells, GRP78 appears to have a tolerogenic role by inhibiting responses to recall antigens and promoting regulatory T- cell formation [41]. Whether extracellular GRP78 exerts a similar or other role in the lung is unstudied.

## Conclusion

Identification of biomarkers which reflect the pathogenic mechanisms underlying the development of cigarette smoke-induced lung injury is an area of intense investigation [5]. GRP78 is crucial to the maintenance of normal lung structure and function and is up-regulated in the setting of lung oxidant stress and lung remodeling [8, 12]. Our present and prior studies indicate that GRP78 secretion into the blood and BALF is increased in the setting of smoking-induced lung injury. These findings suggest the possibility that GRP78 may prove to be a novel biomarker of these processes.

## Abbreviations

BALF: Bronchoalveolar lavage fluid
BEGM: Bronchial epithelial cell growth medium
COPD: Chronic obstructive pulmonary disease
CSC: Cigarette smoke condensate
CSE: Cigarette smoke extract
ER: Endoplasmic reticulum
FEV1: Forced expiratory volume in the first second
FVC: Forced vital capacity
GRP78: Glucose-regulated protein of 78kD
HAEC: Human airway epithelial cells
HDAC: Histone deacetylase
TG: Thapsigargin
